# Clinically practical pharmacometrics computer model to evaluate and personalize pharmacotherapy in pediatric rare diseases: application to Graves' disease

**DOI:** 10.3389/fmed.2023.1099470

**Published:** 2023-05-03

**Authors:** Britta Steffens, Gilbert Koch, Pascal Gächter, Fabien Claude, Verena Gotta, Freya Bachmann, Johannes Schropp, Marco Janner, Dagmar l'Allemand, Daniel Konrad, Tatjana Welzel, Gabor Szinnai, Marc Pfister

**Affiliations:** ^1^Pediatric Pharmacology and Pharmacometrics, University Children's Hospital Basel UKBB, University of Basel, Basel, Switzerland; ^2^Pediatric Endocrinology and Diabetology, University Children's Hospital Basel UKBB, University of Basel, Basel, Switzerland; ^3^Department of Mathematics and Statistics, University of Konstanz, Konstanz, Germany; ^4^Division of Pediatric Endocrinology, Diabetology and Metabolism, Department of Pediatrics, Inselspital, Bern University Hospital, University of Bern, Bern, Switzerland; ^5^Department of Pediatric Endocrinology and Diabetology, Children's Hospital of Eastern Switzerland, St. Gallen, Switzerland; ^6^Division of Pediatric Endocrinology and Diabetology and Children's Research Centre, University Children's Hospital Zurich, University of Zurich, Zurich, Switzerland; ^7^Department of Clinical Research, University of Basel and University Hospital Basel, Basel, Switzerland

**Keywords:** Graves' disease (GD), pediatric rare diseases, hyperthyroidism, thyrotoxicosis, carbimazole monotherapy, block-and-replace therapy, mathematical model, pharmacometrics model

## Abstract

**Objectives:**

Graves' disease (GD) with onset in childhood or adolescence is a rare disease (ORPHA:525731). Current pharmacotherapeutic approaches use antithyroid drugs, such as carbimazole, as monotherapy or in combination with thyroxine hormone substitutes, such as levothyroxine, as block-and-replace therapy to normalize thyroid function and improve patients' quality of life. However, in the context of fluctuating disease activity, especially during puberty, a considerable proportion of pediatric patients with GD is suffering from thyroid hormone concentrations outside the therapeutic reference ranges. Our main goal was to develop a clinically practical pharmacometrics computer model that characterizes and predicts individual disease activity in children with various severity of GD under pharmacotherapy.

**Methods:**

Retrospectively collected clinical data from children and adolescents with GD under up to two years of treatment at four different pediatric hospitals in Switzerland were analyzed. Development of the pharmacometrics computer model is based on the non-linear mixed effects approach accounting for inter-individual variability and incorporating individual patient characteristics. Disease severity groups were defined based on free thyroxine (FT4) measurements at diagnosis.

**Results:**

Data from 44 children with GD (75% female, median age 11 years, 62% receiving monotherapy) were analyzed. FT4 measurements were collected in 13, 15, and 16 pediatric patients with mild, moderate, or severe GD, with a median FT4 at diagnosis of 59.9 pmol/l (IQR 48.4, 76.8), and a total of 494 FT4 measurements during a median follow-up of 1.89 years (IQR 1.69, 1.97). We observed no notable difference between severity groups in terms of patient characteristics, daily carbimazole starting doses, and patient years. The final pharmacometrics computer model was developed based on FT4 measurements and on carbimazole or on carbimazole and levothyroxine doses involving two clinically relevant covariate effects: age at diagnosis and disease severity.

**Discussion:**

We present a tailored pharmacometrics computer model that is able to describe individual FT4 dynamics under both, carbimazole monotherapy and carbimazole/levothyroxine block-and-replace therapy accounting for inter-individual disease progression and treatment response in children and adolescents with GD. Such clinically practical and predictive computer model has the potential to facilitate and enhance personalized pharmacotherapy in pediatric GD, reducing over- and underdosing and avoiding negative short- and long-term consequences. Prospective randomized validation trials are warranted to further validate and fine-tune computer-supported personalized dosing in pediatric GD and other rare pediatric diseases.

## Introduction

Graves' disease (GD) is an autoimmune form of acquired hyperthyroidism (1–5). GD with onset in childhood or adolescence is a rare pediatric disease (ORPHA:525731) in contrast to the adult age group (6, 7). Incidence in children ranges from 0.1 to 3.4/100,000 in Europe and is increasing over decades (Denmark 1982–1988 vs. 1998–2012, 0.79 to 1.58/100,000; Sweden 1990–1999 vs. 2000–2009, 1.6 to 2.8/100,000) (8–11). In three pediatric studies, 71–83% of patients were diagnosed at the age of 10 years or later, 15–20% between 5 and 9 years, and 2–9% were younger than 5 years (8, 12, 13). Children with GD suffer from weight loss, goiter, tachycardia, and tremor at diagnosis, while endocrine orbitopathy is present in ≈30% of affected patients in contrast to a higher frequency of 60–70% in the adult population (4, 5, 9, 13, 14). Anxiety, depression, fatigue, and impaired cognitive function may be associated symptoms and lead to a decline in academic and athletic performance (15–17). Thus, children with GD require adequate, personalized therapy to normalize thyroid function, improve quality of life, and avoid negative long-term consequences as in adults (18, 19).

Antithyroid drugs are the first-line treatment in children with GD (3–5, 20, 21). A definitive cure by thyroidectomy or radioiodine ablation is recommended in patients whose hyperthyroidism is not controlled despite the highest doses of antithyroid drugs or who suffer severe adverse events related to pharmacotherapy (3). Long-term pharmacotherapy with carbimazole (CMZ) is safe before thyroidectomy or radioiodine ablation in children and adolescents (22–24). Pharmacotherapy with propylthiouracil is no longer recommended in pediatric GD due to the increased risk of liver failure (3, 20, 21, 25, 26).

The main goal of pharmacotherapy is normalization of free thyroxine (FT4) levels within 4–8 weeks, depending on disease severity (3, 21, 26, 27). In pediatric GD, two treatment approaches currently exist. The first approach is a complete blocking of thyroid hormone production with CMZ combined with a thyroid hormone substitution with levothyroxine (LT4), also called CMZ/LT4 block-and-replace therapy (3, 20, 21, 26, 28). However, with this treatment approach, up to 25% of pediatric patients show dose-related side effects such as rash and pruritus, and rarely agranulocytosis, hepatitis, and/or vasculitis. For this reason, current international guidelines recommend another treatment approach, a carefully titrating CMZ monotherapy (20, 26, 29, 30). A first recent randomized controlled trial in children with GD showed that there is no evidence for better biochemical control when administering a mean CMZ dose of 0.61 mg/kg/day for CMZ/LT4 block-and-replace vs. 0.3 mg/kg/day for CMZ monotherapy (28). In our retrospective cohort, data show that both treatment approaches were still applied in clinical practice.

Both treatment approaches require frequent outpatient visits and blood sample collections to monitor safety, including clinical and laboratory adverse events of CMZ, and efficacy, to avoid both overdosing associated with iatrogenic hypothyroidism and underdosing resulting in persistent hyperthyroidism (3, 20, 21, 26). Once thyroid hormones are normalized, continuous CMZ adjustments, taking into account the patient's age and weight, disease severity at diagnosis, and disease activity during treatment, are mandatory (3, 20, 21, 26). Disease severity may be extremely variable in patients with newly diagnosed GD. Prepubertal children may, in particular, have severe forms of GD and an increased risk of disease recurrence despite adequate CMZ treatment and appropriate drug adherence (3, 12, 26).

To mitigate the risk of somatic, metabolic, and cognitive impairment, aforementioned aspects should be taken into account for optimized individual CMZ dosing in pediatric GD. To this end, a tailored pharmacometrics (PMX) computer model was developed to characterize FT4 dynamics under both current treatment approaches, CMZ monotherapy and CMZ/LT4 block-and-replace therapy, with the ultimate goal of personalizing CMZ dosing in pediatric GD, (i) reducing the time needed to restore thyroid homeostasis after diagnosis, (ii) increasing the time within reference ranges of thyroid hormones during follow-up, (iii) reducing the number of outpatient visits and blood draws, (iv) enhancing the quality of life, and academic and athletic performance in pediatric patients, and (v) avoiding negative long-term consequences.

For successful implementation in clinical practice, such a tailored PMX computer model (i) has to be in a reasonable balance between complexity to account for physiological mechanisms and simplicity to utilize sparse clinical and laboratory data, and a small number of input parameters (31), (ii) has to follow pharmacokinetics/pharmacodynamics (PK/PD) modeling principles (32–35), and (iii) has to be developed in the context of non-linear mixed effects (NLME) modeling (36).

This research article has two main objectives: first, to perform a descriptive analysis of a retrospective dataset with clinical and laboratory data from children and adolescents with GD up to 2 years of treatment; and second, to develop a clinically practical PMX computer model that can characterize and predict individual FT4 dynamics under CMZ monotherapy and CMZ/LT4 block-and-replace therapy in pediatric GD.

## Methods

This section consists of six paragraphs describing (i) study design and retrospective data collection procedure, (ii) descriptive data analysis of retrospective data of the whole patients' cohort and per disease severity group, (iii) PK and PD components of the PMX computer model for CMZ monotherapy and CMZ/LT4 block-and-replace therapy, (iv) the NLME approach to characterize each individual in a patient population, (v) covariate testing in the PMX modeling process, and (vi) statistical data presentation and applied software packages.

### Study design and retrospective data collection procedure

In this retrospective multicenter longitudinal cohort study of pediatric patients, patient data were included, if (i) children had a confirmed GD [based on elevated FT4 and/or triiodothyronine (T3) and positive antithyroid stimulating hormone receptor antibodies (TRABs)], (ii) children were treated for GD between 1990 and (a) 12/2020 at each of University Children's Hospital Basel, University Hospital Bern, and University Children's Hospital Zurich or (b) 12/2013 at Children's Hospital Eastern Switzerland in St. Gallen, (iii) a complete set of data that includes documented CMZ, clinical baseline characteristics, relevant laboratory parameters at diagnosis and/or start of CMZ treatment, and, in case of CMZ/LT4 block-and-replace therapy, LT4 dose history during the complete follow-up period was available, and (iv) at least two follow-up visits after CMZ treatment start were documented. Pediatric patients were excluded from the study if CMZ and/or LT4 doses were missing at the first visit or any follow-up visit, or if there was documented, inappropriate drug adherence.

Ethical approval for this study (2018-01770) was obtained and amended (01/2021) by the lead local ethics committee (Ethikkommission Nordwest- und Zentralschweiz EKNZ) and all locally responsible ethics committees (Kantonale Ethikkommission Bern, Ethikkommission Zürich, Ethikkommission Ostschweiz EKOS). Data were captured and standardized for each study visit in the designated electronic database secuTrial^®^. Data from patients were pseudonymized. The study was performed in compliance with the tenets of the Declaration of Helsinki and Good Clinical Practice.

### Descriptive analysis of retrospective data

Relevant disease-related clinical and laboratory data, such as age (years), body weight (kilogram, kg), FT4 (pmol/l), and thyroid stimulating hormone (TSH, mU/l), from the start of CMZ treatment (*t*_0_ = 0 day) were included in the analysis. Disease severity was defined based on FT4 measurement at the time of diagnosis [or, if missing, based on TSH and/or thyroxine (T4) measurements]. Patients were categorized as severe (FT4 > 70 pmol/l), moderate (FT4 50–70 pmol/l), and mild (FT4 <50 pmol/l) according to Léger et al. (26).

Total daily CMZ dose (mg/day) and total daily dose per kg body weight (mg/kg/day) were compared between patients receiving CMZ monotherapy and those receiving CMZ/LT4 block-and-replace therapy at the start of LT4 replacement treatment.

Missing daily body weight values were calculated by linear interpolation.

### PK and PD components of the PMX computer model

The prodrug CMZ has a high bioavailability (90–100%) and is quickly absorbed (within 15–30 min) from the gastrointestinal tract (37). After oral administration, CMZ (molecular weight, *MW*_*CMZ*_ = 186.23 g/mol) is rapidly metabolized into the pharmacologically active metabolite methimazole (MMZ) (*MW*_*MMZ*_ = 114.17 g/mol) (37). Since MW_MMZ_/MW_CMZ_ = 0.61, a dose of 1 mg CMZ corresponds to 0.61 mg MMZ. By blocking the thyroid peroxidase enzyme, and hence preventing iodination and coupling of thyroglobulin residues, MMZ reduces the production of the thyroid hormones T3 and T4. The time of maximum MMZ plasma concentration *t*_max_ is reported to be 1–2 h after oral administration of CMZ (37). The elimination half-life *t*_*half*_ of MMZ has been described as variable, ranging from 4 to 12 h, depending on the individual patient, but not related to thyroid status or disease severity (37–40). In addition, in adults, weight dependence of the volume of distribution *V* is referred to as 0.5 l/kg (37), and a bi-exponential profile of MMZ concentration was observed (39).

A general PMX computer model consists of two components: a PK and a PD component. PMX approaches are increasingly utilized to inform key decisions such as dose selection in drug research and development (41, 42). During the last years, PMX models have also been applied to evaluate and optimize dosing strategies in clinical practice, particularly in pediatrics (43–46).

In the context of GD, the PK component characterizes MMZ concentration caused by the administered CMZ doses (Component I), and the PD component characterizes the inhibitory effect of MMZ on the increased endogenous T4 production (Component II). For patients receiving CMZ/LT4 block-and-replace therapy, the administration of LT4 substitutes is included in Component II. Due to this structure, the PMX computer model is explicitly controlled by the doses and is based on pharmacological, physiological, and biological principles.

#### Component I: PK computer model for CMZ and its active metabolite

**Figure 1:**
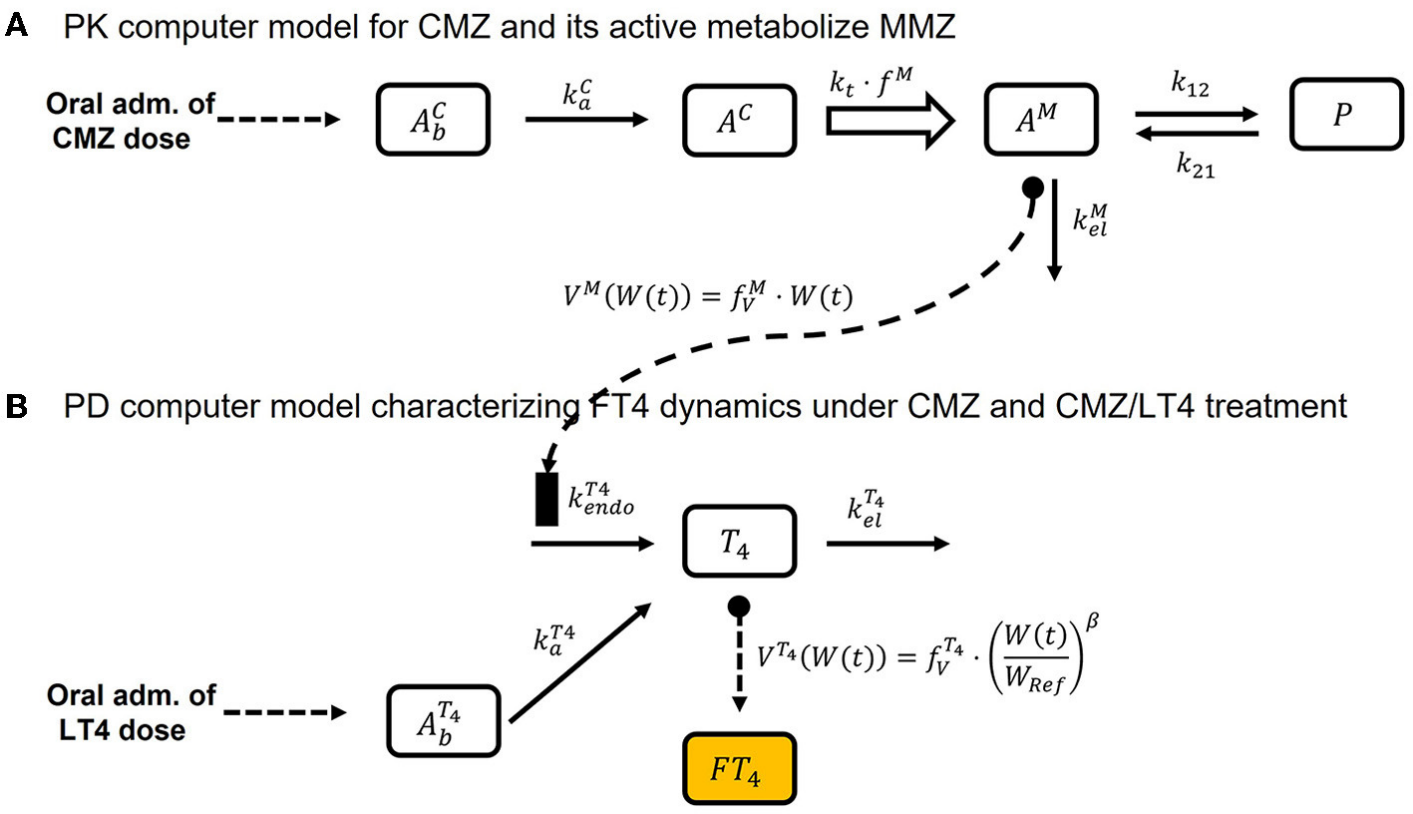
Schematic of the final PMX computer model, Equations (1)–(8). **(A)** Illustrates the schematic of the PK computer model, Equations (1)–(5), and **(B)** shows the schematic of the PD computer model, Equations (6)–(8).

The PK of CMZ and its active metabolite, MMZ, is characterized by a linear multi-compartment model including absorption, metabolism, and peripheral distribution. A schematic of the PK model is shown in Figure 1A, and model equations are presented in the following:

CMZ absorption is characterized by


(1) $$\begin{array}{l} \frac{d}{dt}A_{b}^{C}\left(t\right)=In^{C}\left(t_{j}^{C},d_{j}^{C}\right)-k_{a}^{C}\cdot A_{b}^{C}\left(t\right),\;A_{b}^{C}\left(0\right)=0 \end{array}$$

where $k_{a}^{C}$ (1/day) is the absorption rate and *In*^*C*^ is the dosing input function with a dose of CMZ $d_{j}^{C}$ (mg) and dosing time point $t_{j}^{C}$ (day). The amount of CMZ *A*^*C*^ (mg) in the central compartment is described by


(2) $$\begin{array}{l} \frac{d}{dt}A^{C}\left(t\right)=k_{a}^{C}\cdot A_{b}^{C}\left(t\right)-k_{t}\cdot A^{C}\left(t\right),\;A^{C}\left(0\right)=0 \end{array}$$

where *k*_*t*_ (1/day) is the metabolic transit rate, and the amount of MMZ *A*^*M*^ (mg) in the central compartment is given by


(3) $$\begin{array}{l} \frac{d}{dt}A^{M}\left(t\right)\;=f^{M}\cdot k_{t}\cdot A^{C}\left(t\right)-k_{el}^{M}\cdot A^{M}\left(t\right)-k_{12}\cdot A^{M}\left(t\right)\\ \;+\;k_{21}\cdot P\left(t\right),\;A^{M}\left(0\right)=0\; \end{array}$$

(4) $$\begin{array}{lll} \frac{d}{dt}P\left(t\right) & = & k_{12}\cdot A^{M}\left(t\right)-k_{21}\cdot P\left(t\right),\;P\left(0\right)=0\; \end{array}$$

where *f*^*M*^ (unit-less) is the metabolic conversion factor, $k_{el}^{M}$ (1/day) is the elimination rate, and *k*_12_ (1/day) and *k*_21_ (1/day) are the distribution rates for the peripheral compartment *P*.

Finally, the concentration of MMZ is obtained by


(5) $$\begin{array}{l} C^{M}\left(t\right)=\frac{A^{M}\left(t\right)}{V^{M}\left(W\left(t\right)\right)}\;\text{with }V^{M}\left(W\left(t\right)\right)=f_{V}^{M}\cdot W\left(t\right)\; \end{array}$$

where *W*(*t*) (kg) is the body weight over time and $f_{V}^{M}$ (l/kg) is a proportionality factor relating current body weight with volume of distribution of MMZ.

#### Component II: PD computer model characterizing FT4 dynamics under treatment

The structural PMX computer model for endogenous T4 production and exogenous LT4 treatment is similar to the one-compartment PK model for congenital hypothyroidism presented by Koch et al. (31), but with one difference in interpretation: in the case of CMZ/LT4 block-and-replace therapy, $k_{endo}^{T4}$ (nmol/day) denotes the (GD-induced) increased endogenous T4 production rate. Under CMZ monotherapy, this endogenous T4 production will be inhibited. This means that the proposed PMX computer model extends the model presented by Koch et al. (31) by including the inhibitory effect of CMZ treatment on the endogenous T4 production rate *via* the MMZ concentration.

To be precise, utilizing a commonly used inhibitory effect term (32, 34, 47) on $k_{endo}^{T4}$ (nmol/day) with respect to *C*^*M*^(*t*) (mg/l) results in


(6) $$\begin{array}{lll} \frac{d}{dt}A_{b}^{T4}\left(t\right) & = & In^{T4}\left(t_{j}^{T4},d_{j}^{T4},F^{T4}\right)-k_{a}^{T4}\cdot A_{b}^{T4}\left(t\right),\;A_{b}^{T4}\left(0\right)=0\; \end{array}$$

(7) $$\begin{array}{l} \frac{d}{dt}T4\left(t\right)=k_{a}^{T4}\cdot A_{b}^{T4}\left(t\right)+k_{endo}^{T4}\cdot \left(1-\frac{I_{\max}\cdot C^{M}\left(t\right)}{IC_{50}+C^{M}\left(t\right)}\right)\\ \;-k_{el}^{T4}\cdot T4\left(t\right),\;T4\left(0\right)=\frac{k_{endo}^{T4}}{k_{el}^{T4}}\; \end{array}$$

(8) $$\begin{array}{lll} C^{FT4}\left(t\right) & = & \frac{0.3\cdot T4\left(t\right)}{V^{T4}\left(W\left(t\right)\right)}\;\text{with }V^{T4}\left(W\left(t\right)\right)=f_{V}^{T4}\cdot {\left(\frac{W\left(t\right)}{W_{Ref}}\right)}^{\beta }\; \end{array}$$

where *I*_max_ ≤ 1 (unit-less) is the maximal inhibitory drug effect, *IC*_50_ (mg/l) is the MMZ concentration causing the half-maximal inhibitory effect, *In*^*T*4^ is the dosing input function, $d_{j}^{T4}$ (mcg/day) is the dose of LT4 at the time point $t_{j}^{T4}$ (day) converted with factor 1.29 to nmol/day (*MW*_*T*4_ = 776.9 g/mol), *F*^*T*4^ ≤ 1 (unit-less) is the bioavailability of LT4, and $k_{a}^{T4}$ (1/day) is the respective absorption rate. The initial condition for T4 in Equation (7) ensures that the endogenous T4 production is in equilibrium before treatment, as per Koch et al. (31). Finally, FT4 concentration was obtained by converting the T4 unit nmol/l into the FT4 unit pmol/l, and assuming that the FT4 concentration corresponds to 0.03% of T4, as per Koch et al. (31). Weight dependence of the volume of distribution *V*^*T*4^ (l) was included in an analogous manner as in Koch et al. (31). This means that *V*^*T*4^ is assumed to be proportional to a power of the quotient of current weight and reference weight *W*_*Ref*_ (corresponding to this GD population) with multiplicative factor $f_{V}^{T4}$ (l) and power exponent β (unit-less). A schematic of Equations (6)–(8) is presented in Figure 1B.

The final PMX computer model consists of Equations (1)–(8), and the entire schematic is displayed in Figure 1. It should be noted, in the case of CMZ monotherapy, that *In*^*T*4^ is equal to zero for all *t*. Hence, $A_{b}^{T4}$ is also zero.

Time-varying covariates were tested as additional regressors and directly implemented in the PMX computer model (Equations 1–8) as proposed in the Monolix Suite 2021R1 (Lixoft, Orsay, France). All time-constant covariates were tested with the Monolix Suite 2021R1's default settings.

### NLME approach to characterize each individual in a patient population

NLME modeling (36) is the gold standard to simultaneously characterize individual patients stemming from the same cohort/population. The basic idea is that, on the one hand, each patient shares some similar properties, e.g., physiology and disease, but on the other hand, has individual distinctive characteristics, e.g., response to treatment. Hence, the final PMX computer model (Equations 1–8) is structurally valid for all patients in the population, but each patient has their own individual model parameters. The result of the NLME analysis consists of (i) population model parameters (fixed effects) characterizing the average patient in the population, (ii) standard deviations (random effects) characterizing the distributions of the individual model parameters, and (iii) effects of covariates on model parameters. Application of the NLME analysis is of major importance to developing a predictive PMX computer model that, in clinical practice, can be reliably applied to a newly diagnosed patient since this patient will not only be classified based on its available FT4 measurements and its covariates but also on the knowledge learned from the previously analyzed patients' cohort/population represented by fixed and random effects.

### Covariate testing in the PMX modeling process

Covariates to be tested in the PMX computer model were selected based on completeness, possible correlations among each other, and clinical plausibility. We included age at diagnosis, sex, and disease severity in the covariate testing. In addition, the type of treatment in terms of CMZ monotherapy or CMZ/LT4 block-and-replace therapy was included as a possible categorical covariate.

The continuous covariates TSH, weight, and body mass index at diagnosis were not available for all patients and were therefore not tested. Body weight over time, with interpolated measurements for missing values, was already included in the model for scaling volume of distribution, comparing Equations (5) and (8).

We also tested the effect of changing the type of treatment on the endogenous T4 production rate $k_{endo}^{T4}$ by implementing an on/off switch depending on whether the patient received CMZ only or both CMZ and LT4.

TSH effects over time were not tested for two reasons. First, based on the available data, TSH did not provide more information about the observed FT4 variability than was already described by the model. Second, regular TRAB measurements are not required during follow-up and thus were not tested as a possible covariate.

### Statistical data presentation and applied software packages

All laboratory and demographic values are reported as median together with the interquartile range (IQR) (25%-percentile, 75%-percentile). Descriptive statistical analysis and simulations were performed in R 4.1.0 (R core team, Vienna, Austria), and hypothesis testing was executed with the Student's *t*-test for normally distributed values and with the Wilcoxon rank sum test for non-normally distributed values. We consider a *p*-value smaller than 0.05 to be significant. NLME modeling was performed in the Monolix Suite 2021R1. A-posteriori data visualization was implemented in R or MATLAB 2020a (MathWorks, Natick, MA, USA).

## Results

The Results section consists of three paragraphs, describing: (i) retrospective data, (ii) parameter setup of the PK and PD components of the final PMX computer model and covariate effects, and (iii) additional sensitivity analyses.

### Descriptive analysis of retrospective data

A total of 58 pediatric patients with GD were found to be eligible. After screening for inclusion and exclusion criteria, 44 patients (75% female) were included for modeling and further analysis. All these patients had more than two FT4 measurements, and body weight information (*n* = 455) was available from at least 30% of clinical visits during follow-up. According to the severity grading defined by Léger et al. (26), 13 patients were categorized as mild, 15 patients as moderate, and 16 patients as severe.

Patient characteristics at diagnosis such as age (years), weight (kg), FT4 (pmol/l), and TSH (mU/l), in addition to total daily CMZ starting dose (mg/day) and total daily CMZ starting dose per kg body weight (mg/kg/day) per disease severity group, were compared. We observed no significant difference among the three groups in terms of patient characteristics, total daily CMZ doses, and patient years and follow-up years.

**Figure 2:**
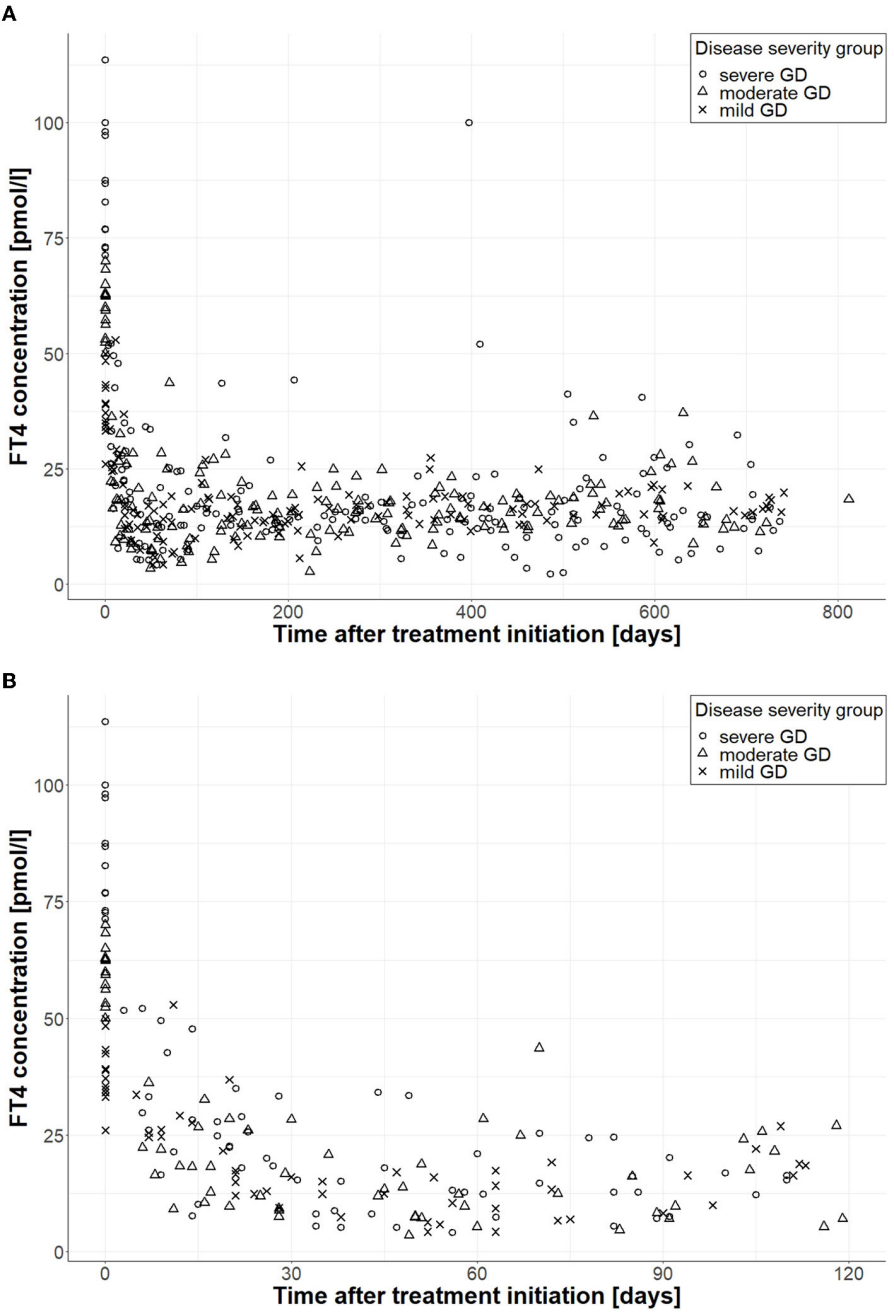
FT4 measurements according to disease severity during follow-up. **(A)** Shows FT4 measurements during the whole follow-up (*n* = 494), and **(B)** displays FT4 measurements during the first 120 days (*n* = 129); circles correspond to FT4 measurements of patients with severe GD, triangles belong to patients with moderate GD, and crosses correspond to patients with mild GD.

**Figure 3:**
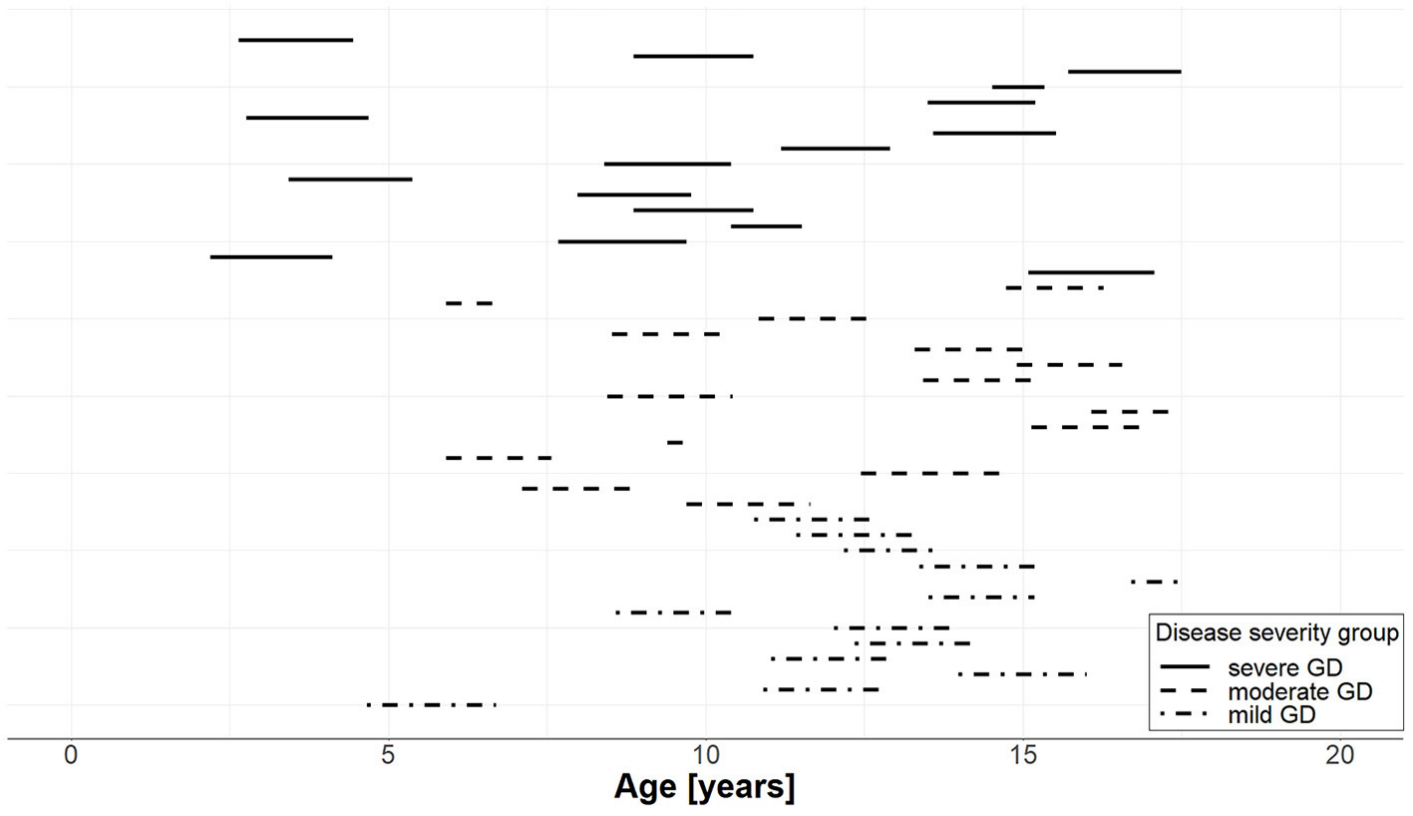
Years of follow-up for each of the 44 individual patients with GD per severity group. The start of the line represents the age at the start of pharmacotherapy and the length of the line represents the duration of follow-up; solid lines correspond to patients with severe GD, dashed lines to patients with moderate GD, and dash-dotted lines to patients with mild GD.

On average, 11 FT4 measurements were available per patient with a minimum of 3 and a maximum of 36 measurements. The median end of monitoring was at day 688.5 (617.8, 719.0) with a range of 126 to 812 days. FT4 measurements (*n* = 494) of the final patient cohort divided by severity group during the whole follow-up (Panel A) and during the first 120 days (Panel B) are shown in Figure 2, and the years of follow-up for each patient are displayed in Figure 3.

A total of *n* = 27 patients (61%) received CMZ monotherapy. For *n* = 17 patients receiving CMZ/LT4 block-and-replace therapy, the median start of LT4 treatment, and thus the median start of CMZ/LT4 block-and-replace therapy, was at day 75 (52, 183) ranging from 28 to 364 days.

Regarding the two current pharmacotherapy approaches, we observed no significant difference in patient characteristics at diagnosis, total daily CMZ starting doses, and total daily CMZ starting doses per kg body weight. The median total daily CMZ starting dose per kg body weight was 0.70 (0.60, 0.77) mg/kg/day with a range of [0.36, 1.35] mg/kg/day for patients receiving CMZ monotherapy, and for CMZ/LT4 block-and-replace therapy, 0.76 (0.60, 0.87) mg/kg/day with a range of [0.37, 1.40] mg/kg/day.

Detailed information on patient characteristics at the time of diagnosis is presented in Table 1, and information regarding treatment during follow-up is presented in Table 2.

**Table 1:** Patient characteristics at baseline for all patients and by disease severity, expressed as n (%) for categorical variables or as median (IQR) for continuous variables.

	**All patients**	**Patients with severe GD^a^**	**Patients with moderate GD^a^**	**Patients with mild GD^a^**	**Stats severe moderate mild**
		**FT4 > 70 pmol/l**	**FT4 50–70 pmol/l**	**FT4 <50 pmol/l**	
Patients (*n*)^b^	44	16 (36%)	15 (34%)	13 (30%)	
Female patients (*n*)	33 (75%)	15 (94%)	10 (67%)	8 (62%)	
Age (yrs)	11.0 (8.4, 13.5)	8.9 (6.6, 13.5)	10.8 (8.5, 14.1)	12.0 (10.9, 13.4)	n.s.^f^
Weight (kg)^c^	31.4 (27.8, 50.0)	31.9 (28.6, 50.0)	30.5 (25.7, 39.1)	41.5 (27.9, 46.2)	n.s.^f^
FT4 (pmol/l)^d^	59.9 (48.4, 76.8)	84.8 (76.9, 97.9)	59.6 (53.9, 62.9)	38.9 (34.8, 43.2)	
TSH (mU/l)^e^	0.002 (0.002, 0.004)	0.002 (0.004, 0.006)	0.002 (0.002, 0.002)	0.002 (0.002, 0.003)	

a Disease severity defined according to guidelines (13).

b Three missing values for FT4 at diagnosis, severity groups determined by TSH and/or T4 measurements.

c Six missing values.

d Three missing values.

e One missing value.

f Not significant if *p*-value ≥ 0.05.

**Table 2:** Patient information on treatment at baseline and during follow-up for all patients and by disease severity, expressed as n (%) for categorical variables or as median (IQR) for continuous variables.

	**All patients**	**Patients with severe GD^a^**	**Patients with moderate GD^a^**	**Patients with mild GD^a^**	**Stats severe moderate mild**
		**FT4 >70 pmol/l**	**FT4 50–70 pmol/l**	**FT4 <50 pmol/l**	
Patient years (yrs)	76.44	28.27	24.43	23.74	
Follow-up (yrs)	1.89 (1.69, 1.97)	1.89 (1.77, 1.94)	1.79 (1.59, 1.91)	1.97 (1.88, 1.99)	n.s.^c^
CMZ starting dose (mg/day)	19.28 (19.28, 38.56)	20.18 (19.28, 38.56)	19.28 (19.28, 32.13)	19.28 (19.28, 32.13)	
CMZ starting dose per kg of body weight (mg/kg/day)^b^	0.72 (0.6, 0.81)	0.73 (0.65, 0.81)	0.67 (0.55, 0.83)	0.7 (0.45, 0.77)	n.s.^c^
Patients receiving CMZ monotherapy (*n*)	27 (61%)	11 (69%)	10 (67%)	6 (46%)	
Time point of switch to block-and-replace therapy (days after treatment initiation)	75 (52, 183)	85 (43, 89)	119 (60, 254)	73 (52, 75)	n.s.^c^
LT4 starting dose for block-and-replace therapy (mcg/kg/day)^b^	1.86 (1.57, 2.20)	1.78 (1.69, 2.17)	2.12 (1.57, 2.20)	1.86 (1.34, 2.22)	n.s.^c^
CMZ dose at switch to block-and-replace therapy (mg/kg/day)^b^	0.42 (0.32, 0.44)	0.43 (0.33, 0.53)	0.43 (0.32, 0.44)	0.38 (0.30, 0.42)	n.s.^c^

a Disease severity defined according to guidelines (13).

b Computed based on calculated values for missing weight measurements.

c Not significant if *p*-value ≥ 0.05.

### PK and PD components of the PMX computer model

#### Component I: Computer PK model for CMZ and its active metabolite

The PK model parameters of CMZ and its active metabolite MMZ (Equations 1–5) were based on values reported in the literature (39, 40) and product labels (37). To realize a peak time of *t*_max_ = 2 (h) for MMZ concentration after oral CMZ administration, $k_{a}^{C}$ was set to 144 (1/day) and *k*_*t*_ to 28.8 (1/day). The half-life of MMZ was assumed to be 6 h, and therefore $k_{el}^{M}$ was set to 2.77 (1/day). The metabolic conversion factor was set to *f*^*M*^ = 0.61 (unit-less), and the distribution rates *k*_12_ and *k*_21_ for the peripheral compartment were both set to 2.4 (1/day). The proportionality factor relating body weight to the volume of distribution was reported as $f_{V}^{M}=0.5$ (l/kg). In our population, CMZ was administered three times a day and was implemented accordingly for data fitting and parameter estimation.

**Figure 4:**
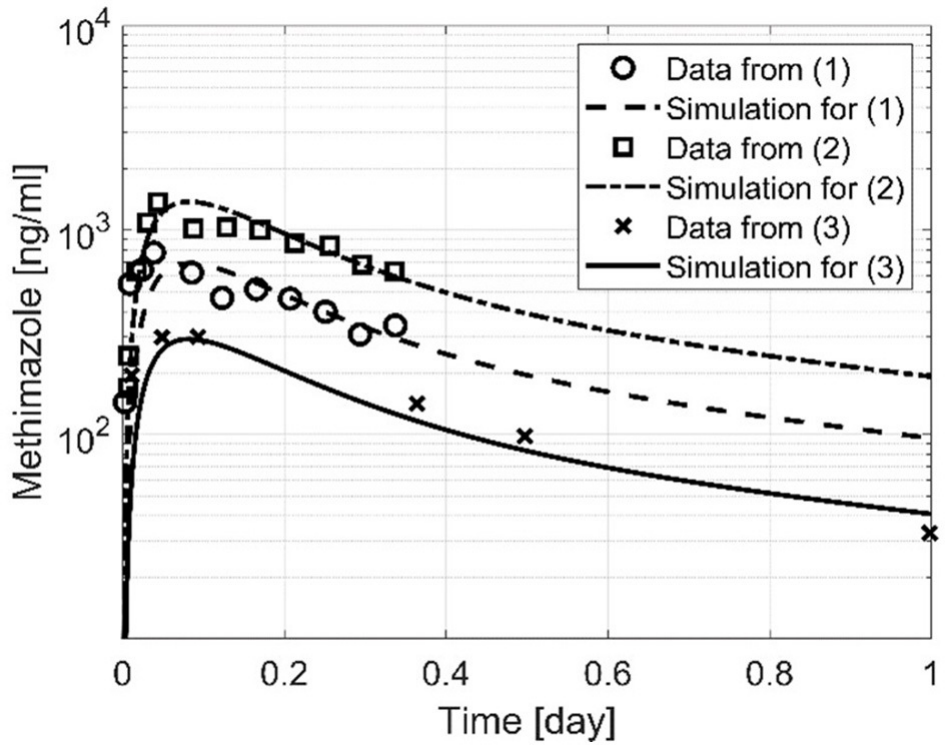
Three simulations based on the developed PK computer model, Equations (1)–(5). MMZ measurements for (1) and (2) were obtained from Cooper et al. (48) with an oral dose of 30 and 60 mg, and MMZ measurements for (3) were obtained from Okamura et al. (39) with an oral dose of 10 mg.

To demonstrate the predictive capability of the developed PK model (Equations 1–5) for CMZ and its active metabolite MMZ, average MMZ concentrations of hyperthyroid patients were digitized from Cooper et al. (48) and Okamura et al. (39) for a single oral MMZ administration of 10, 30, and 60 mg. To apply our PK model, MMZ doses were converted to CMZ doses, resulting in 16.4, 49.2, and 96.4 mg. In Cooper et al. (48), MMZ was measured after oral MMZ administration of 30 and 60 mg in five hyperthyroid GD patients for up to 8 h. Since weight was not reported, we assumed an average value of 60 kg. In Okamura et al. (39), MMZ was measured after oral MMZ administration of 10 mg in 15 hyperthyroid patients for up to 48 h with an average weight of 46.8 kg. Please compare Figure 4 for measurements and simulation of the CMZ PK model Equations (1)–(5) (the figure was reduced to 1 day for clarity). Overall, the developed PK model (Equations 1–5) characterized the digitized data from the literature well with the chosen parameter values.

#### Component II: PD computer model characterizing FT4 dynamics under treatment

FT4 measurements were fitted with the NLME approach (36, 49). In brief, each individual has the same structural model (Equations 1–8); however, individual covariate effects on model parameters are implemented, and most model parameters follow a (usually log-normal) distribution to realize inter-individual variability (IIV).

The bioavailability of LT4 was set to *F*^*T*4^ = 0.6 in Equation (6), and the reference weight value *W*_*Ref*_ was chosen as the median for all patients in this GD population over the entire treatment duration, resulting in *W*_*Ref*_ = 42.7 kg. The T4 absorption rate $k_{a}^{T4}\;$in Equations (6)–(7) was set to $k_{a}^{T4}$ = 20 (1/day) and the T4 elimination rate $k_{el}^{T4}$ was set to $k_{el}^{T4}$ = 0.1 (1/day), both without IIV, as per Koch et al. (31) for rationale. In our population, LT4 was administered once per day. The endogenous T4 production rate $k_{endo}^{T4}$ (nmol/day) and the MMZ concentration causing the half-maximal inhibitory effect *IC*_50_ (mg/l) in Equation (7) were estimated with IIV. With regard to Equation (8), both the factor $f_{V}^{T4}$ (l) relating body weight with the volume of distribution of T4 and the power exponent β (unit-less) were estimated with a fixed standard deviation for the IIV. Interestingly, the estimated population value of β was close to the value obtained in neonates and infants with congenital hypothyroidism, compared to Koch et al. (31). Finally, the maximum inhibitory drug effect parameter in Equation (7) was set to *I*_max_ = 0.9 without IIV. A log-normal distribution was applied to all parameters equipped with IIV.

Significant covariate effects of disease severity and age at diagnosis on the endogenous production rate $k_{endo}^{T4}$ (nmol/day) were found and included in the final PMX computer model. Almost all of the IIV could be explained by these covariate effects.

The final covariate effect model for an individual's endogenous T4 production rate $k_{endo,i}^{T4}$ is


$$\begin{array}{l} \log\left(k_{endo,i}^{T4}\right)=\log\left(k_{endo}^{T4}\right)+\beta_{k_{endo}^{T4}}^{AGE}\cdot \log\left(\frac{AGE}{AGE_{Ref}}\right)\\ \;+\;\{\begin{array}{c} \;0\text{ for mild GD }\\ \beta_{k_{endo}^{T4}}^{Cat\;2}\text{ for moderate GD}\\ \beta_{k_{endo}^{T4}}^{Cat\;1}\text{ for severe GD} \end{array} \end{array}$$

where *AGE*_*Ref*_ = 8.98 (yrs) corresponds to the weighted mean, the default setting in the Monolix Suite.

All model parameter estimates and values are shown in Table 3. Goodness-of-fit plots and a selection of individual profiles are presented in the Supplementary material.

**Table 3:** Population estimates (fixed effects), standard deviation of random effects, covariate effect parameters, and additional parameters obtained from data fitting using the final PMX computer model, Equations (1)–(8).

**Parameter**	**Description**	**Unit**	**Estimate (r.s.e.^a^)**
**Population estimates (fixed effects)**			
$k_{a}^{C}$	CMZ absorption rate	1/day	144 fix
*k*_*t*_	Metabolism transit rate	1/day	28.8 fix
*f* ^*M*^	Metabolic conversion factor	unit-less	0.61 fix
$k_{el}^{M}$	MMZ elimination rate	1/day	2.77 fix
*k*_12_	Peripheral compartment distribution rate	1/day	2.4 fix
*k*_21_	Peripheral compartment distribution rate	1/day	2.4 fix
$f_{V}^{M}$	Proportionality factor	l/kg	0.5 fix
$k_{a}^{T4}$	T4 absorption rate	1/day	20 fix
$k_{endo}^{T4}$	Endogenous T4 production rate	nmol/day	261 (7)
$k_{el}^{T4}$	T4 elimination rate	1/day	0.1 fix
*I*_max_	Maximal inhibitory effect of MMZ	unit-less	0.9 fix
*IC*_50_	MMZ concentration for half-maximal inhibitory effect	mg/l	0.024 (18)
$f_{V}^{T4}$	Multiplicative factor	l	20.3 (9)
β	Power exponent	unit-less	0.7 (18)
**Standard deviation of the random effects**			
$\omega_{k_{endo\;}^{T4}}$			0.04 (58)
ω_*I*_*C*__50__			0.77 (15)
$\omega_{f_{V}^{T4}}$			0.1 fix
ω_*beta*_			0.25 fix
**Covariate effect parameters**			
$\beta_{k_{endo}^{T4}}^{AGE}$	Age effect on $k_{endo}^{T4}$		0.464 (13)
$\beta_{k_{endo}^{T4}}^{Cat\;1}$	Disease severity (severe GD) effect on $k_{endo}^{T4}$		0.52 (14)
$\beta_{k_{endo}^{T4}}^{Cat\;2}$	Disease severity (moderate GD) effect on $k_{endo}^{T4}$		0.16 (49)
**Additional parameters**			
Prop. res. error			0.344 (4)
−2LL value			3,333

a Relative standard error.

The rationale for the fixed population parameters is found in the Results section.

### Additional sensitivity analyses

In the following, additional sensitivity analyses are reported for completeness. We explicitly emphasize that these results are not part of the final PMX computer model and solely serve as additional information.

#### Evaluation of the potential effect of CMZ/LT4 block-and-replace therapy on the endogenous T4 production

We tested whether the type of treatment, i.e., receiving CMZ monotherapy vs. receiving CMZ/LT4 block-and-replace therapy, has an impact on thyroid function and inhibition during follow-up. In the case of CMZ/LT4 block-and-replace therapy, a reduced endogenous thyroid function, represented by a smaller value for $k_{endo}^{T4}$ (nmol/day) and a reduced half-maximal inhibitory effect, represented by a smaller value for *IC*_50_ (mg/l), were observed. As there is no obvious clinically reasonable interpretation for these potential effects, further investigation is warranted.

#### Application of a simplified PK computer model for CMZ treatment and its active metabolite

The applied PK computer model for CMZ treatment and its active metabolite MMZ (Equations 1–5) is a very detailed and mechanism-based representation. However, due to several numerical and technical reasons, such a detailed representation can be computationally extremely elaborate. Hence, we tested whether a simplified PK computer model leads to similar results regarding data fitting, parameter estimates, and goodness-of-fit plots.

Due to the fast absorption of CMZ, the quick metabolism after CMZ administration, and the three times daily drug administration, it is possible to replace Equations (1)–(5) by a simplified one-compartment intravenous (IV) model with a single total dose per day:


(9) $$\begin{array}{l} \frac{d}{dt}\bar{A^{M}}\left(t\right)=In^{C}\left(\bar{t_{k}^{C}},\bar{d_{k}^{C}},F^{C}\right)-k_{el}^{M}\cdot \bar{A^{M}}\left(t\right),\;\bar{A^{M}}\left(0\right)=0 \end{array}$$

(10) $$\begin{array}{l} \bar{C^{M}}\left(t\right)=\frac{\bar{A^{M}}\left(t\right)}{V^{M}\left(W\left(t\right)\right)}\;with\;V^{M}\left(W\left(t\right)\right)=f_{V}^{M}\cdot W\left(t\right) \end{array}$$

where $\bar{d_{k}^{C}}$ (mg) is the total dose per day at the time point $\bar{t_{k}^{C}}$ (day) and the value of the metabolic conversion factor is now incorporated in the input function *In*^*C*^*via* the scaling factor *F*^*C*^. The remaining parameters $k_{el}^{M}$ and $f_{V}^{M}$ have been chosen as before.

The profile of $\bar{C^{M}\;}\left(t\right)$ Equations (9) and (10) has higher but daily peak concentrations and therefore impacts the value of the half-maximal inhibitory effect caused by the MMZ concentration *IC*_50_. As expected, the simplified PK model Equations (9) and (10) produced a lower *IC*_50_ value of 0.016 (mg/l). All other parameters, the objective function value, and the goodness-of-fit plots showed no significant difference.

A major advantage of the simplified PK model Equations (9) and (10) is the reduction of computational costs, not only during data fitting but also for potential application in a clinical setting. The PMX computer model with the simplified CMZ PK Equations (6)–(10) is approximately six times faster than the PMX computer model with the detailed CMZ PK Equations (1)–(8). Hence, if no PK measurements are available for data fitting, and a large difference exists in the time scales of CMZ treatment (hours) and FT4 measurements (weeks, months), a simplified PK model performs equally well.

## Discussion

In this section, we discuss the main results in the context of our two research objectives: (i) descriptive analysis of retrospective clinical data from 44 pediatric patients with mild, moderate, or severe GD, and (ii) development of a clinically practical PMX computer model that can characterize and predict individual FT4 dynamics under both current treatment approaches in pediatric GD (20, 21, 26, 29).

The first objective was a descriptive analysis of this retrospectively collected multicenter data of pediatric patients diagnosed with GD. We included 44 pediatric patients with equal numbers per severity group. In this GD cohort, two treatment approaches were observed. The majority (61%) received CMZ monotherapy, whereas all other patients (39%) received CMZ/LT4 block-and-replace therapy with a median start of LT4 administration at day 75 (IQR 52, 183). Although hepatic insufficiency is known as a contraindication for the continuing administration of CMZ, in this retrospective study, hepatic values were not documented systematically. However, in the case of moderate hepatitis, treatment would be stopped, and, as reported in a recent systematic review (50), mild elevation of transaminases is rare in children (1% of treated pediatric patients).

The general challenges of collecting patient records with a rare disease and the limitations of retrospective multicenter studies were discussed in Koch et al. (31). In addition, the challenges and opportunities of developing a clinically relevant PMX computer model for congenital hypothyroidism (CH) based on retrospectively collected multicenter data are explained (31). To harmonize the data, we introduced a time-dependent normalizing method. However, in this study, compared to the CH-study (31), we observed two decisive distinctions: (i) age-specific laboratory FT4 reference ranges showed only slight and negligible differences, and (ii) information about laboratory reference ranges was incomplete for a few patients. Since, as already discussed in Koch et al. (31), normalization should be considered “*the last resort”* (51), we did not apply a normalizing method for potential laboratory differences in this analysis.

The second objective was to develop a practical and predictive PMX computer model to characterize FT4 dynamics under CMZ monotherapy and CMZ/LT4 block-and-replace therapy for clinical application. Since the 1950s, several mathematical models describing the hypothalamic–pituitary–thyroid axis have been developed, offering detailed insights into the complexity of the underlying physiological mechanisms. However, most of these publications focus on a specific research question and not on applicability and feasibility in clinical practice (31, 52–62). Our final, tailored PMX computer model (Equations 1–8, 6–10) requires minimal data, which are typically measured during outpatient visits. In addition, it was developed in the NLME framework, accounts for the typical pharmacological, physiological, and biological principles, and allows for individualized therapy. In this NLME context, not only the FT4 measurements and covariates of a newly diagnosed patient are utilized for prediction but also all the learned knowledge from the already analyzed representative population.

The presented clinically practical PMX computer model consists of a detailed PK model for CMZ treatment and its active metabolite MMZ based on information from the literature. We demonstrated that this detailed PK model well-describes the MMZ concentration digitized from the literature. However, since MMZ concentration is not measured in clinical practice, we showed that the complex PK computer model (Equations 1–5) can be replaced by a simpler PK computer model (Equations 9–10) which may be of great advantage in clinical application by reducing the required input parameters and computational cost. In addition, we have shown that the use of the simplified PK computer model only affects the *IC*_50_ value but does not affect the quality of the data fitting.

Moreover, we observed two clinically relevant covariate effects on the endogenous T4 production rate. On the one hand, we observed a positive effect of age at diagnosis. From a clinical perspective, this effect can be explained by the size of the thyroid. The older the patients at diagnosis, the larger the volume of the thyroid, and consequently, the larger the endogenous T4 production rate. On the other hand, we observed an effect of disease severity on the endogenous production rate. The three disease severity levels, mild, moderate, and severe GD, were defined based on FT4 at diagnosis, and increased FT4 values correspond to a larger endogenous T4 production rate. Using the patients with mild GD as the reference group, the severity effect is positive, resulting in an increased endogenous production rate for more severely diseased patients. In detail, this means, for patients with moderate GD, the endogenous production rate is, with respect to the production rate of mild diseased patients, increased by <20%, and for patients with severe GD by more than 65%.

**Figure 5:**
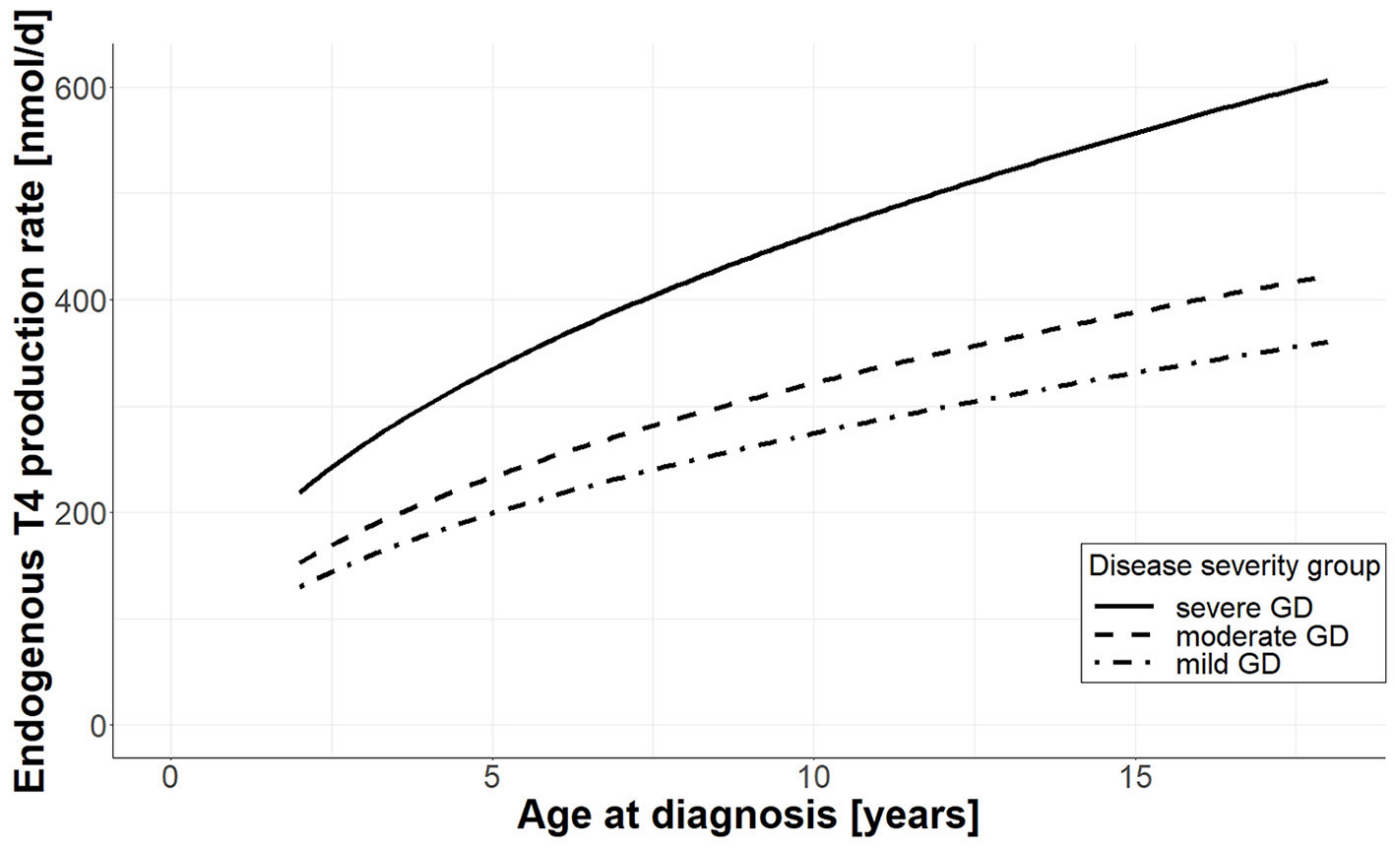
Effect of age and disease severity on endogenous T4 production rate. For each disease severity group, the endogenous production rate (*y*-axis) is plotted as a function of age at diagnosis (*x*-axis); the solid line shows the production rate for patients with severe GD, the dashed line corresponds to patients with moderate GD, and the dashed-dotted line corresponds to patients with mild GD.

In Figure 5, we illustrate both covariate effects by plotting the endogenous T4 production rate (*y*-axis) as a function of age at diagnosis (*x*-axis) for each of the three different disease severity groups, namely severe GD (solid line), moderate GD (dashed line), and mild GD (dashed-dotted line).

Two further aspects are of interest for translation and application in clinical practice. On the one hand, due to the NLME modeling framework, our practical PMX computer model allows for personalized individual dose optimization (63) for pharmacotherapy in pediatric GD and facilitates the implementation of PMX-based clinical decision support tools (64).

On the other hand, as heart rate (HR) is a useful clinical marker to monitor thyroid activity in children with GD under treatment, it would be interesting to test for possible effects when including heart rate values in the model as a covariate. Due to a considerable number of missing values (23%) on tachycardia and heart function at diagnosis in our retrospective dataset, this could not be realized in this PMX model development. We aim to extend our developed PMX computer model by adding an FT4-HR component that describes the relationship between FT4 kinetics and the development of tachycardia.

Recent case reports indicate that wearable devices that continuously measure heart rate may facilitate the diagnosis and monitoring of adolescents with GD during the COVID-19 pandemic (65, 66). Furthermore, a recent prospective longitudinal study demonstrates the feasibility of monitoring heart rate in adults with hyperthyroidism using wearable technology. The combination of heart rate as a clinical marker in addition to FT4 dynamics has the potential to further facilitate the implementation of our predictive PMX computer model in clinical practice (67). As such, the application of our PMX modeling approach to heart rate monitoring at on-site consultation and at home with wearable devices is the obvious next step toward the implementation of computer-assisted, enhanced individualized monitoring and treatment of children and adolescents with GD in clinical practice.

In conclusion, the developed clinically practical PMX computer model with PK and PD components that account for inter-individual disease progression and treatment response is a promising approach to evaluate and improve individualized pharmacotherapy in children with GD with the potential to reduce over- and underdosing and avoid negative short- and long-term consequences. Prospective randomized validation trials are warranted to further validate and fine-tune computer-assisted, personalized pharmacotherapy in pediatric GD and other rare pediatric diseases.

## Data availability statement

The original contributions presented in the study are included in the article/Supplementary material, further inquiries can be directed to the corresponding author.

## Ethics statement

The studies involving human participants were reviewed and approved by Lead local Ethics Committee (Ethikkommission Nordwest- und Zentralschweiz EKNZ) and all local responsible Ethics Committees (Kantonale Ethikkommission Bern, Ethikkommission Zürich, Ethikkommission Ostschweiz EKOS). Written informed consent to participate in this study was provided by the participants' legal guardian/next of kin.

## Author contributions

GK, TW, and GS designed the study protocol. PG, FC, MJ, Dl'A, DK, and GS were responsible for patient recruitment and data collection. BS, GK, GS, and MP analyzed the data. BS, GK, VG, FB, JS, and MP were responsible for or contributed to model development. BS, GK, FC, GS, and MP wrote the manuscript. All authors provided critical feedback on the manuscript and read and approved the submitted version.
