# Supplementary material for: Clinically practical pharmacometrics computer model to evaluate and personalize pharmacotherapy in pediatric rare diseases: application to Graves' disease
Source: Front Med (Lausanne). 2023 May 3;10:1099470. doi: 10.3389/fmed.2023.1099470 (PMC10188966; doi:10.3389/fmed.2023.1099470)
Supplement: Supplementary file 1 [file Data_Sheet_1.PDF]

Supplementary material

The first figure shows goodness-of-fit plots for detailed PMX computer model Eqs. (1)-(8). VPCs indicate good model performance:

(A)

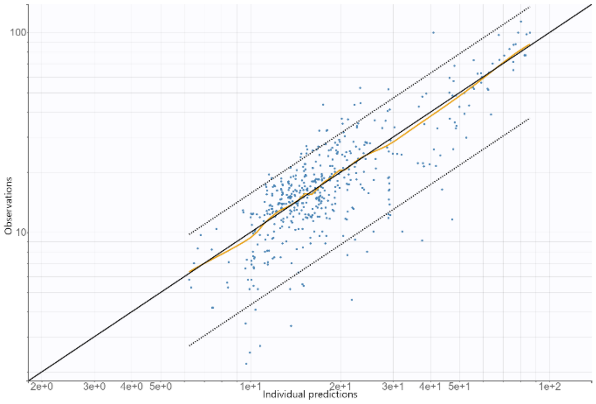

(B)

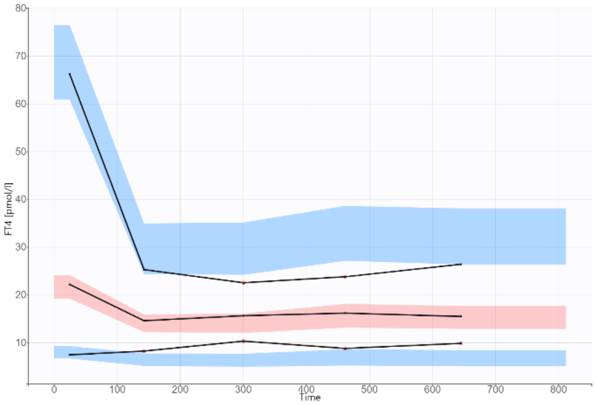

(C)

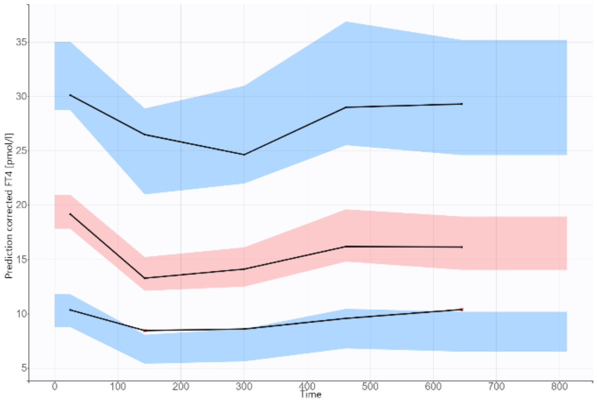

Figure S1: *Goodness-of-fit plots for final PMX computer model: Observation vs. prediction plot in log-log-scale (Panel A), VPC (Panel B), prediction corrected VPC (Panel C).*

The second figure shows a selection of well fitted individual profiles for patients receiving either CMZ monotherapy or CMZ/LT4 block-and-replace therapy. Each profile corresponds to one severity group:

(A)

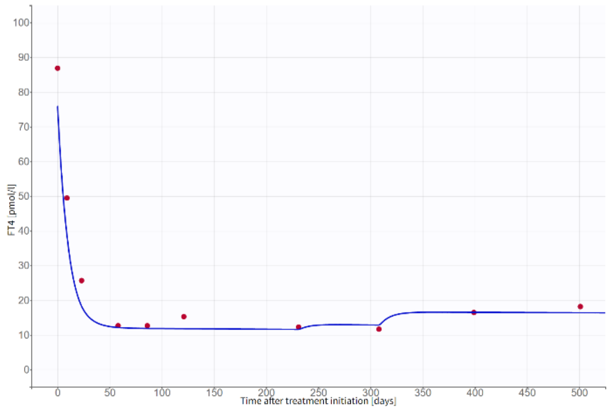

(B)

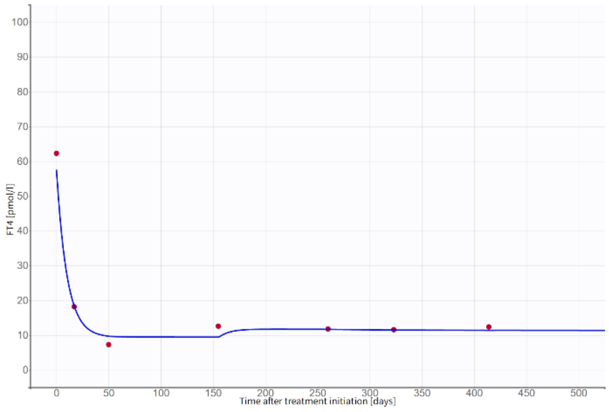

(C)

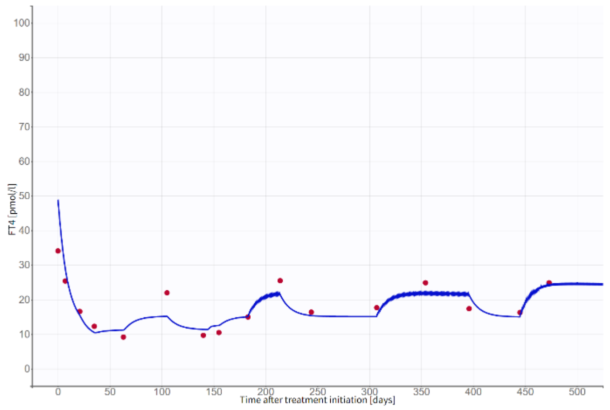

Figure S2: *Individual fits for patients receiving CMZ monotherapy are shown in Panel A (severe GD) and Panel B (moderate GD), and individual fit for a patient receiving CMZ/LT4 block-and-replace therapy is shown in Panel C (mild GD); red dots show measured FT4 concentrations [pmol/l] and blue line display fitted FT4 concentration curve.*
